# The vertical stratification of potential bridge vectors of mosquito-borne viruses in a central Amazonian forest bordering Manaus, Brazil

**DOI:** 10.1038/s41598-020-75178-3

**Published:** 2020-10-26

**Authors:** Adam Hendy, Eduardo Hernandez-Acosta, Danielle Valério, Claudia Mendonça, Edson Rodrigues Costa, José Tenaçol Andes Júnior, Flamarion Prado Assunção, Vera Margarete Scarpassa, Marcelo Gordo, Nelson Ferreira Fé, Michaela Buenemann, Marcus Vinícius Guimarães de Lacerda, Kathryn A. Hanley, Nikos Vasilakis

**Affiliations:** 1 Department of Pathology and Center for Biodefense and Emerging Infectious Diseases, Center for Tropical Diseases, Institute for Human Infections and Immunity, University of Texas Medical Branch, 301 University Blvd, Galveston, TX 77555-0609 USA; 2 Department of Biology, New Mexico State University, 1200 S Horseshoe St, Las Cruces, NM 88003-8001 USA; 3 Fundação de Medicina Tropical Dr Heitor Vieira Dourado (FMT-HVD), Avenida Pedro Teixeira, 25, Dom Pedro, Manaus, AM 69040-000 Brazil; 4 Laboratório de Biologia da Conservação, Projeto Sauim-de-Coleira, Instituto de Ciências Biológicas, Universidade Federal do Amazonas, Avenida General Rodrigo Octávio Jordão Ramos, 6200, Coroado, Manaus, AM 69080-900 Brazil; 5 Laboratório de Genética de Populações e Evolução de Mosquitos Vetores de Malária e Dengue, Coordenação de Biodiversidade, Instituto Nacional de Pesquisas da Amazônia, Avenida André Araújo, 2936, Bairro Aleixo, Manaus, AM 69067-375 Brazil; 6 Department of Geography, New Mexico State University, 1525 Stewart St, Las Cruces, NM 88003-8003 USA

**Keywords:** Entomology, Alphaviruses, Dengue virus, Viral reservoirs, Viral vectors

## Abstract

The emergence of Zika virus (ZIKV) in Latin America brought to the fore longstanding concerns that forests bordering urban areas may provide a gateway for arbovirus spillback from humans to wildlife. To bridge urban and sylvatic transmission cycles, mosquitoes must co-occur with both humans and potential wildlife hosts, such as monkeys, in space and time. We deployed BG-Sentinel traps at heights of 0, 5, 10, and 15 m in trees in a rainforest reserve bordering Manaus, Brazil, to characterize the vertical stratification of mosquitoes and their associations with microclimate and to identify potential bridge vectors. *Haemagogus janthinomys* and *Sabethes chloropterus*, two known flavivirus vectors, showed significant stratification, occurring most frequently above the ground. *Psorophora amazonica*, a poorly studied anthropophilic species of unknown vector status, showed no stratification and was the most abundant species at all heights sampled. High temperatures and low humidity are common features of forest edges and microclimate analyses revealed negative associations between minimum relative humidity, which was inversely correlated with maximum temperature, and the occurrence of *Haemagogus* and *Sabethes* mosquitoes. In this reserve, human habitations border the forest while tamarin and capuchin monkeys are also common to edge habitats, creating opportunities for the spillback of mosquito-borne viruses.

## Introduction

In Latin America, Zika (ZIKV, *Flaviviridae*: *Flavivirus*), dengue (DENV, *Flaviviridae*: *Flavivirus*), and chikungunya (CHIKV, *Togaviridae*: *Alphavirus*) viruses exist in human-endemic cycles of transmission involving *Aedes aegypti* and *Ae. albopictus* mosquitoes^1,2^. However, their ancestral lineages can be traced to the forests of Africa (ZIKV, CHIKV) and Southeast Asia (DENV), where each of these viruses is transmitted in sylvatic cycles among non-human primates by canopy-dwelling *Aedes* species^1^. There is significant concern that ZIKV could spill back into a New World sylvatic cycle, precluding regional elimination and creating an enduring threat to human health^1^. These concerns stem from similarities between the natural histories of yellow fever virus (YFV, *Flaviviridae*: *Flavivirus*) and ZIKV. In Africa, both viruses are maintained among many of the same monkey and mosquito species^3–5^. YFV was introduced to the Americas around 400 years ago^6^, after which it spilled back into a sylvatic cycle involving neotropical monkeys^7^ and *Haemagogus* and *Sabethes* species mosquitoes^8,9^. Recently, some of these same monkey and mosquito species have been shown to be susceptible to experimental infection with ZIKV^10–13^, and ZIKV has been isolated from monkeys in suburban neighborhoods in Brazil^14,15^. However, the virus has not been isolated from free-living forest mosquitoes, although at present, only low-numbers of the most likely sylvatic vector species have been screened^16,17^.

In light of these developments, there is a need to identify the species best poised to act as bridge vectors that could transfer ZIKV from humans to wildlife and back again. In this case, potential bridge vectors are those mosquitoes that are competent for ZIKV infection and bite both humans and potential wildlife reservoir species. The rate of contact between a vector and its host is a crucial component of vectorial capacity^18^, and a successful bridge vector must, therefore, co-occur in space and time with both humans and wildlife reservoirs. To elucidate potential bridge vectors, we previously investigated the distribution of mosquitoes in urban parks in Manaus, Brazil^19^. Manaus is a city of more than 2 million people surrounded by the Amazon rainforest, where abrupt urban-forest edges bring humans and animals into close contact and where DENV, CHIKV, and ZIKV circulate regularly. In these parks, *Ae. albopictus* and *Ae. aegypti* penetrated at least 100 m from the urban edge and may therefore serve as bridge vectors if they feed on both humans and monkeys. *Haemagogus janthinomys, Sabethes glaucodaemon*, and *Sa. tridentatus* were among multiple taxa of forest-dwelling mosquitoes present close to the urban-forest edge, but were rare^19^. However, these mosquitoes were sampled with BG-Sentinel traps placed at ground level, which likely favored the collection of ground-dwelling species and underrepresented the arboreal mosquitoes active at higher heights.

The main vectors of sylvatic YFV in the Americas, *Hg. janthinomys*, *Hg. leucocelaenus*, and *Sa. chloropterus*^20,21^, are arboreal species that all occur in the Amazon. All three are diurnally active mosquitoes inhabiting forest canopies^20^, a behavior termed “acrodendrophily”^22^, that lay eggs in natural containers such as tree holes or bamboo internodes^23,24^. Monkeys are thought to be an important bloodmeal source for these species^25^, although they will opportunistically feed on humans when in close contact^20,26^. There is also serological evidence that *Hg. janthinomys* and *Hg. leucocelaenus* feed on a range of other vertebrates including birds, rodents, and marsupials^27,28^.

Despite their acrodendrophilic habits, *Hg. janthinomys*, *Hg. leucocelaenus*, and *Sa. chloropterus* are not restricted to the forest canopy^20^. Both *Hg. janthinomys* and *Sa. chloropterus* are strongly acrodendrophilic^25, 29–31^, while *Hg. leucocelaenus* shows a more dispersed distribution between the canopy and ground^32,33^. However, there is some evidence that the stratification of *Hg. janthinomys*^32^ and *Sa. chloropterus*^29^ changes seasonally, and there are mainly anecdotal reports that acrodendrophilic species including *Hg. janthinomys* bite at ground level at forest edges^20,34^ and elsewhere where the canopy is disturbed^30,32^. There is also limited quantitative evidence that variation in microclimate may affect the vertical distribution of *Haemagogus* and *Sabethes* mosquitoes, with strongly acrodendrophilic species seeming to prefer hotter and less humid conditions associated with the forest canopy^25,32^. The physiological condition of mosquitoes in general may also influence their preferred activity height, as may external factors such as the distribution of their hosts^35,36^.

In this study, we investigated the composition and stratification of mosquito species at the Adolpho Ducke forest reserve which borders the city of Manaus, focusing on likely bridge vectors of mosquito-borne viruses between urban and sylvatic cycles. We predicted that known or probable bridge vectors would show distributions extending from the forest floor to the highest heights sampled, corresponding with strata most commonly occupied by humans and monkeys. In addition, we predicted that the most strongly acrodendrophilic species would be more abundant at relatively higher temperatures and lower humidity. Finally, we tested associations between 7-day cumulative rainfall in the weeks prior to collections and the occurrence of specific taxa, predicting that they would be consistent with the development times of particular species.

## Methods

### Study area

**Figure 1:**
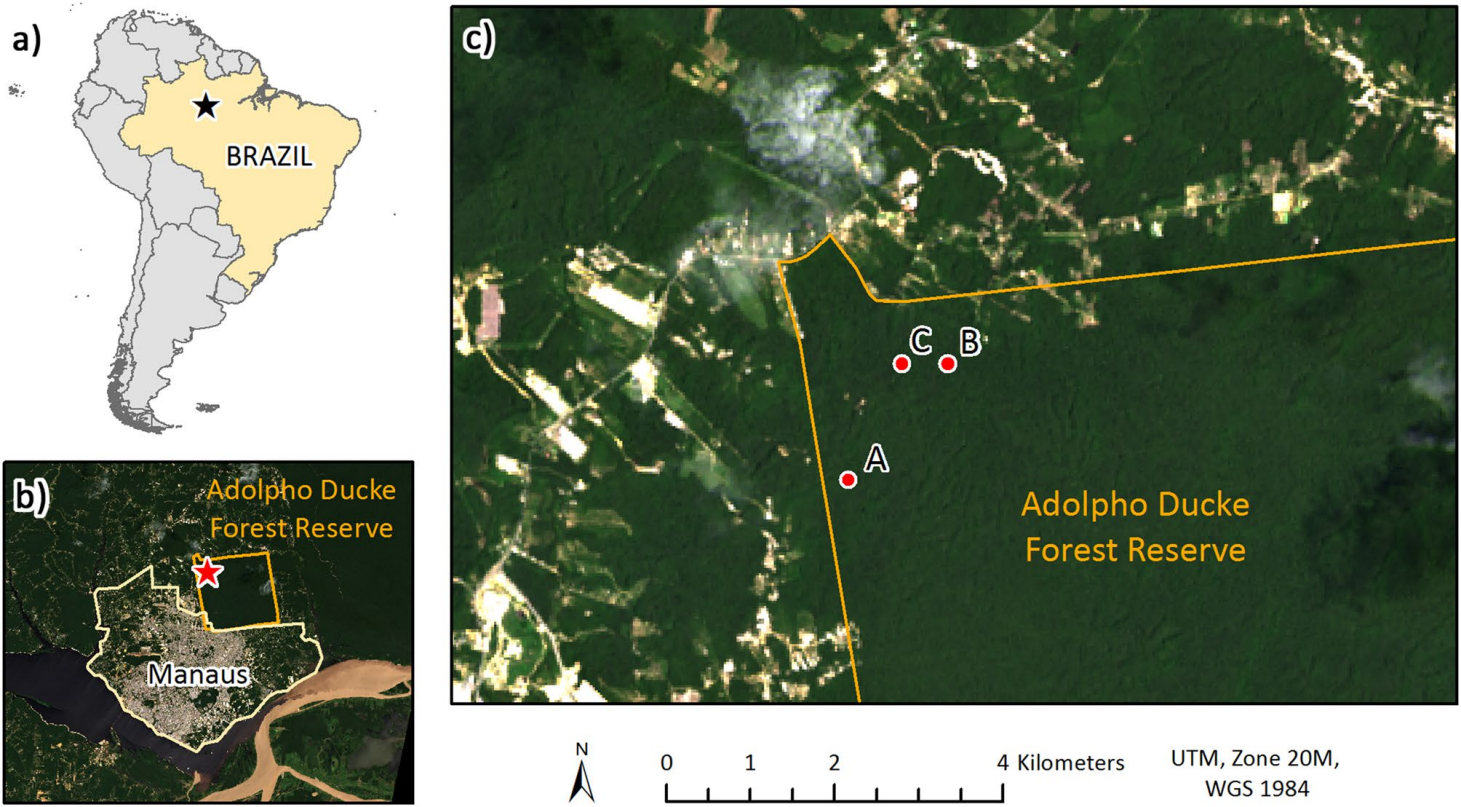
Map showing location of (**a**) Manaus in Brazil, (**b**) the Adolpho Ducke forest reserve in Manaus and study area (star) in the northwest of the reserve, and (**c**) study sites A, B, and C, where collections were made. Orange outline shows the approximate extent of the Ducke reserve. Map created using ArcGIS Desktop 10.7.1 (ESRI, Redlands, California).

**Figure 2:**
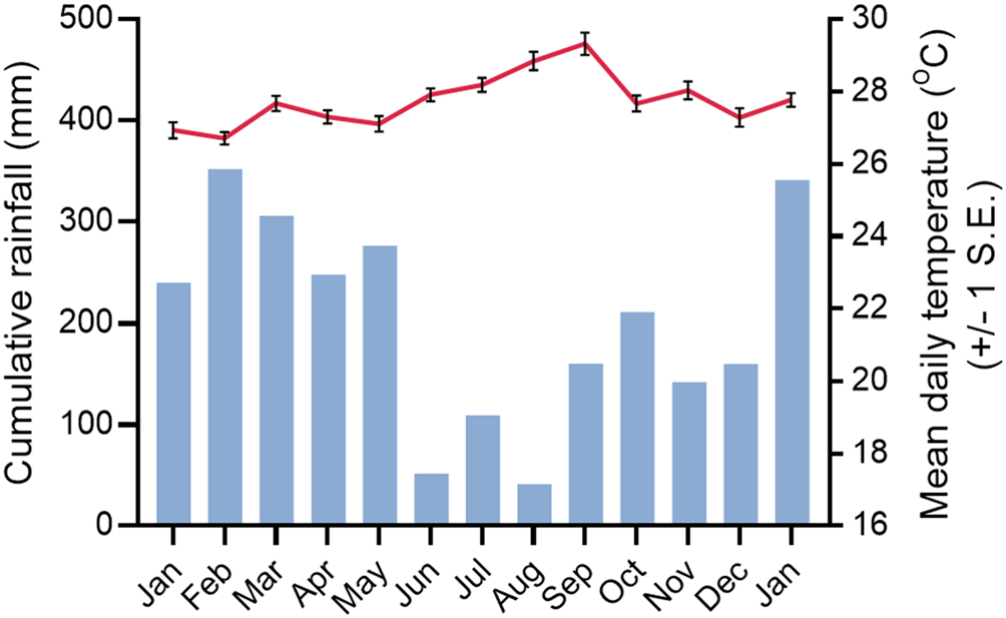
Weather during the study period from January 2019 until January 2020, recorded at the INMET meteorological station in Manaus^39^. Bars show cumulative monthly rainfall (mm) and line shows mean daily temperature per month ± 1 standard error (S.E.).

The Adolpho Ducke forest reserve is 100 km^2^ of primary rainforest^37^ situated north of Manaus in Amazonas State, Brazil (Fig. 1). The reserve borders Manaus in the northeast of the city where the two form an abrupt edge between urban and forest environments. The remaining boundaries border peri-urban or rural areas, which are contiguous with the Amazon rainforest. Canopy height in the region is 30–35 m with emergent trees reaching 50 m, while palms including *Astrocaryum*, *Attalea, Bactris,* and *Geonoma* species are abundant in the low-light understory. Ducke receives relatively light foot-traffic compared to other forest parks in Manaus, being mainly accessed by students and researchers. However, nearby communities, and densely populated urban areas in the southwest bring humans into close contact with the forest and its wildlife. In total, six monkey species have been recorded in the reserve^38^, namely the Guianan red howler monkey (*Alouatta macconnelli*), Guiana spider monkey (*Ateles paniscus*), tufted capuchin (*Sapajus apella*), bearded saki (*Chiropotes chiropotes*), golden-faced saki (*Pithecia chrysocephala*), and pied tamarin (*Saguinus bicolor*). Relative humidity exceeds 70% and annual precipitation ranges from 1750 to 2500 mm. The rainy season lasts from November to May while the dry season lasts from June to October^37^ (Fig. 2).

### Study sites

Three study sites, A (central coordinates: 02.93776° S, 059.97646° W), B (central coordinates: 02.92537° S, 059.96582° W), and C (central coordinates: 02.92531° S, 059.97069° W), were established approximately 500–600 m from the forest edge in the north western corner of the reserve (Fig. 1). Site A was chosen based on prior knowledge of the mosquito fauna at this location, which included *Haemagogus* and *Sabethes* mosquitoes. Distance to forest edge was then controlled as a variable when choosing sites B and C, where these mosquitoes were also found to be present following pilot collections. Both *Haemagogus* and *Sabethes* species are thought to exhibit low dispersal when oviposition sites are abundant^40^ and distances of 500 m–2 km were, therefore, maintained between the sites to minimize spatial autocorrelation while ensuring that they were accessible. Each site was in a section of forest populated by large trees approximately 20–30 m in height and was close to an area of open canopy caused by at least one tree fall.

### BG-Sentinel trap collections in trees

BG-Sentinel 2 traps (BioQuip, Rancho Dominguez, California) baited with CO_2_ (1 kg dry ice) and BG-Lure, a synthetic attractant containing ammonia, lactic acid, and caproic acid, were used to sample mosquitoes at heights of 0, 5, 10, and 15 m. Thirteen trees were sampled in total (Table 1); five at site A, and four at sites B and C. Trees were selected that (1) had appropriately high branches for suspending traps, (2) had either visible tree holes or were thought large enough to permit breeding by target species, (3) were situated within reasonable proximity to treefall gaps, and (4) were concentrated in areas where the mosquitoes of interest were present. For these reasons, the trees were clustered within 75 m at each site, but distances of > 10 m were maintained between them to minimize trap interference^41^. In each tree, a fishing line was placed over a branch using a Big Shot (SherrillTree, Greensboro, North Carolina) slingshot (Supplementary Fig. S1). The line was then substituted with a rope marked at 1 m intervals. A BG-Sentinel trap was tied to the rope and equipped with dry ice, which was wrapped in four sheets of newspaper and placed inside a sealable plastic bag with a 1 cm hole in the corner, before being raised to the desired height.

**Table 1:** Identity and measurement of trees in which traps were placed at each site.

Site	Family	Genus	Species	Local name	DBH (cm)
A	*Elaeocarpaceae*	*Sloanea*	*guianensis*	Urucurana	30.3
	*Lecythidaceae*	*Eschweilera*	*coriacea*	Matamatá-branco	99.4
	*Lecythidaceae*	*Eschweilera*	*pedicellata*	Castanha Vermelha	49.4
	*Melastomataceae*	*Mouriri*	*angulicosta*	Muiraúba	67.2
	*Sapotaceae*	*Pouteria*	*cladantha*	Abiurana	52.7
B	*Apocynaceae*	*Geissospermum*	*argenteum*	Acariquara-branca	52.5
	*Fabaceae*	*Dipteryx*	*polyphylla*	Cumarurana	68.5
	*Lecythidaceae*	*Eschweilera*	*pseudodecolorans*	Ripeiro	90.4
	*Lecythidaceae*	*Eschweilera*	*truncata*	Matamatá	75.2
C	*Chrysobalanaceae*	*Licania*	*rodriguesii*	Pajurazinho	68.8
	*Fabaceae*	*Pseudopiptadenia*	*psilostachya*	Faveira-folha-fina	35.0
	*Lecythidaceae*	*Corythophora*	*alta*	Ripeiro Vermelho	75.8
	*Sapotaceae*	*Micropholis*	*guyanensis*	Risadinha	46.5

DBH (cm) = diameter at breast height (1.3 m) in centimeters. See Supplementary file: Dataset  for cross reference between tree species and mosquito collections.

When collecting at a site, one trap was placed in each tree at a randomly selected elevated height (5, 10, or 15 m) and one trap was placed at ground level (0 m) at the base of one or two of the trees. Traps were generally set before 11:00 and left to run for approximately 24 h before mosquitoes were collected. Different heights were then randomly selected, without replacement, for subsequent collections until all elevated and ground level heights had been sampled. The process was then repeated. The study design combined with occasional battery failure meant that there were days when no ground-level traps were running, or fewer traps were running at elevated heights, but overall, an equal number of collections were made at each of the four heights at each site. Collections started at site A on 24 January 2019 at the beginning of the rainy season (Fig. 2), followed by site B on 7 March and site C on 18 March. Once all sites had been established, collections were rotated between the three in a 3 × 3 Latin square design. From 17 June, all three sites were sampled simultaneously to increase the yield of target species before collections were suspended on 27 June 2019 at the beginning of the dry season when the abundance of target species diminished. Simultaneous collections were then restarted at sites B and C on 11 November 2019 following a period of substantial rainfall (Fig. 2), which was twice the monthly mean for September and October^42^, and collections continued until 9 January 2020.

### Sample storage and permits

All mosquitoes were stored in the field on dry ice and were stored in the laboratory at the Fundação de Medicina Tropical Doutor Heitor Vieira Dourado at − 80 °C until identified. Permission to collect mosquitoes was provided by the Brazilian Ministry of the Environment (SISBIO 57003-6).

### Environmental variables

In order to investigate variation in microclimate among heights and associations between microclimate and mosquito distributions, temperature (°C) and relative humidity (%) were recorded at 30-min intervals using Hygrochron iButton data loggers (Maxim Integrated, San Jose, California) placed in a catch bag (BioQuip, Rancho Dominguez, California) on the outside of each trap. These data were used to calculate the minimum, maximum, mean, daytime mean (06:00–18:00), and range of both variables. In addition, precipitation data were obtained from the automated INMET meteorological station (Code: Manaus-A101, OMM: 81730) located in the city of Manaus (3.103682° S, 60.015461° W) and were used to calculate several precipitation variables. These were defined as total rain (cumulative rainfall in mm during the ≈24 h sampling period), early rain (cumulative rainfall from 08:00 to 13:00 on the day traps were set), late rain (cumulative rainfall from 14:00 to 17:00 on the day traps were set), and daytime rain (cumulative rainfall from 08:00 to 17:00 on the day traps were set). Seven-day cumulative rainfall was also calculated at lags of 1, 2, 3, and 4 weeks prior to each collection to test associations between past rainfall and the occurrence of specific taxa.

### Mosquito identifications

Individual mosquitoes were placed on a chill table (BioQuip, Rancho Dominguez, California) and were identified to genus and, where possible, species using a stereomicroscope and relevant taxonomic keys^43–49^. Genus and species names follow nomenclature in the Walter Reed Biosystematics Unit New Mosquito Classification^50^. All samples were stored at − 80 °C for future arbovirus screening.

### Statistical analyses

Microclimate data were not normally distributed and variation in temperature and humidity parameters among heights was, therefore, analyzed using Kruskal–Wallis tests followed by post-hoc Wilcoxon Each Pair tests to compare differences between heights. Linear regression with site (A, B, or C) as the unit of replication was used to test the effect of height on the mean number of identified species and species diversity as measured by the Shannon–Wiener diversity index using the Vegan package in R v3.6.1^51^. Two approaches were taken to compare mosquito communities at different heights: first, the Morisita overlap index was calculated to compare mosquito community overlap between collection sites and between sampled heights using PAST: Paleontological statistics software package^52^, second, a principal components analysis of relative frequencies of each species was conducted followed by hierarchical clustering of communities at each height. Due to the low abundance of *Hg. janthinomys* and *Sa. chloropterus*, Pearson’s chi-squared tests followed by post-hoc two-tailed Fisher’s exact tests were used to investigate the number of traps positive at each height for target species with large sample sizes. The latter test was also used to compare the number of traps positive at ground level with those above the ground when sample sizes were small. Pairwise Spearman’s rank analyses were used to investigate correlations between trap height, temperature, humidity, and rainfall variables except for rainfall lag, and nominal logistic regressions were used to investigate the impact of target variables on the occurrence of designated mosquito taxa at each height. Nominal logistic regressions were also used to test associations between rainfall lag and the occurrence of designated taxa. Statistical analyses were performed using JMP 14^53^ unless otherwise stated.

## Results

### Variation in microclimate among forest strata

**Figure 3:**
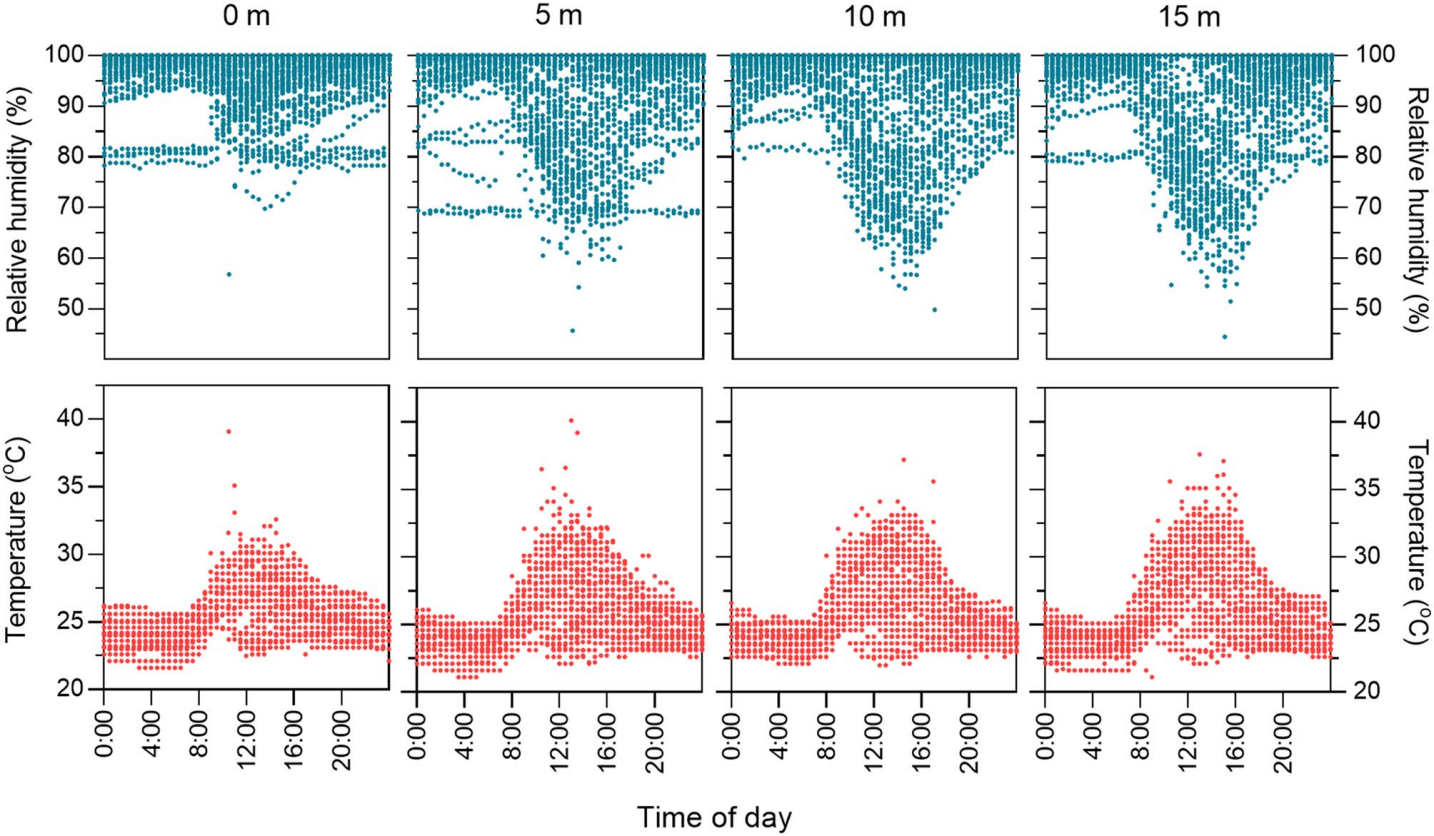
Daily variation in microclimate among forest strata showing increasing temperature (°C) and decreasing relative humidity (%) with increasing height. Data recorded at 30-min intervals for duration of sampling.

**Figure 4:**
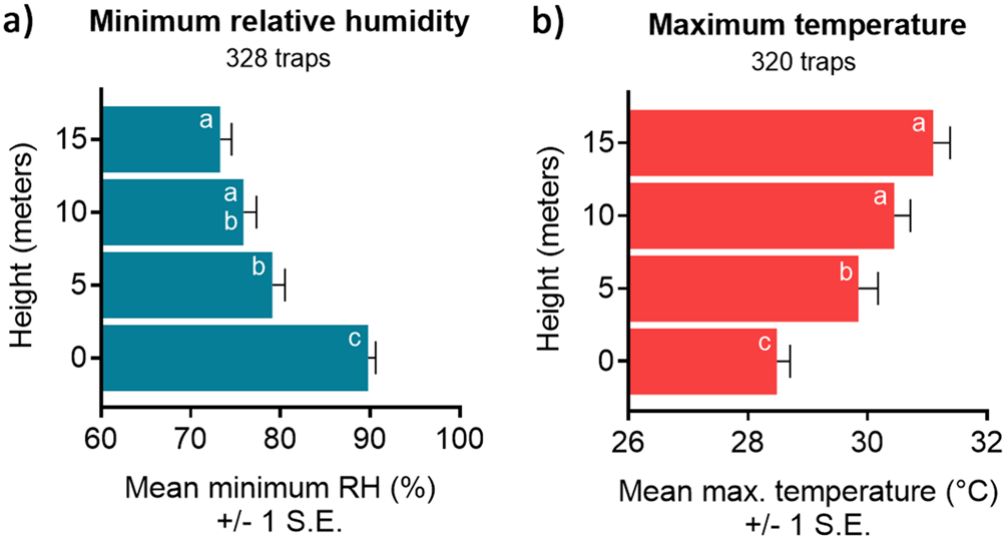
Variation in microclimate showing (**a**) mean minimum relative humidity (%) and (**b**) mean maximum temperature (°C) ± 1 standard error (S.E.) at each height sampled. Minimum relative humidity based on observations from 328 traps and maximum temperature based on observations from 320 trap collections. Different letters within bars indicate significant differences in occurrence between heights for each species; letters indicate within-panel comparisons only.

As expected, temperatures rose, and humidity fell with increasing height above the forest floor (Fig. 3). Spearman’s rank analyses showed that almost all temperature, humidity, and rainfall variables were significantly correlated (*P* < 0.05) with one another (Supplementary Table S1). Temperature and humidity variables, apart from maximum humidity (*P* = 0.57), were also significantly correlated with trap height, but rainfall and trap height were not correlated. There was a significant effect of height on minimum relative humidity (Kruskal–Wallis, DF = 3, χ^2^ = 75.6, *P* < 0.0001) and maximum temperature (DF = 3, χ^2^ = 55.8, *P* < 0.0001) (Fig. 4), which were chosen as two key variables that together captured variation in microclimate. Post-hoc Wilcoxon Each Pair analyses (exact *P* values listed in Supplementary Table S2) revealed that these differences were significant (P < 0.05) when comparing 0 m with all elevated heights for both variables; when comparing 5 m with 10 m for maximum temperature only; and when comparing 5 m with 15 m for both variables. However, differences were not significant for either variable when comparing 10 m with 15 m.

### Relative abundance of mosquitoes

**Figure 5:**
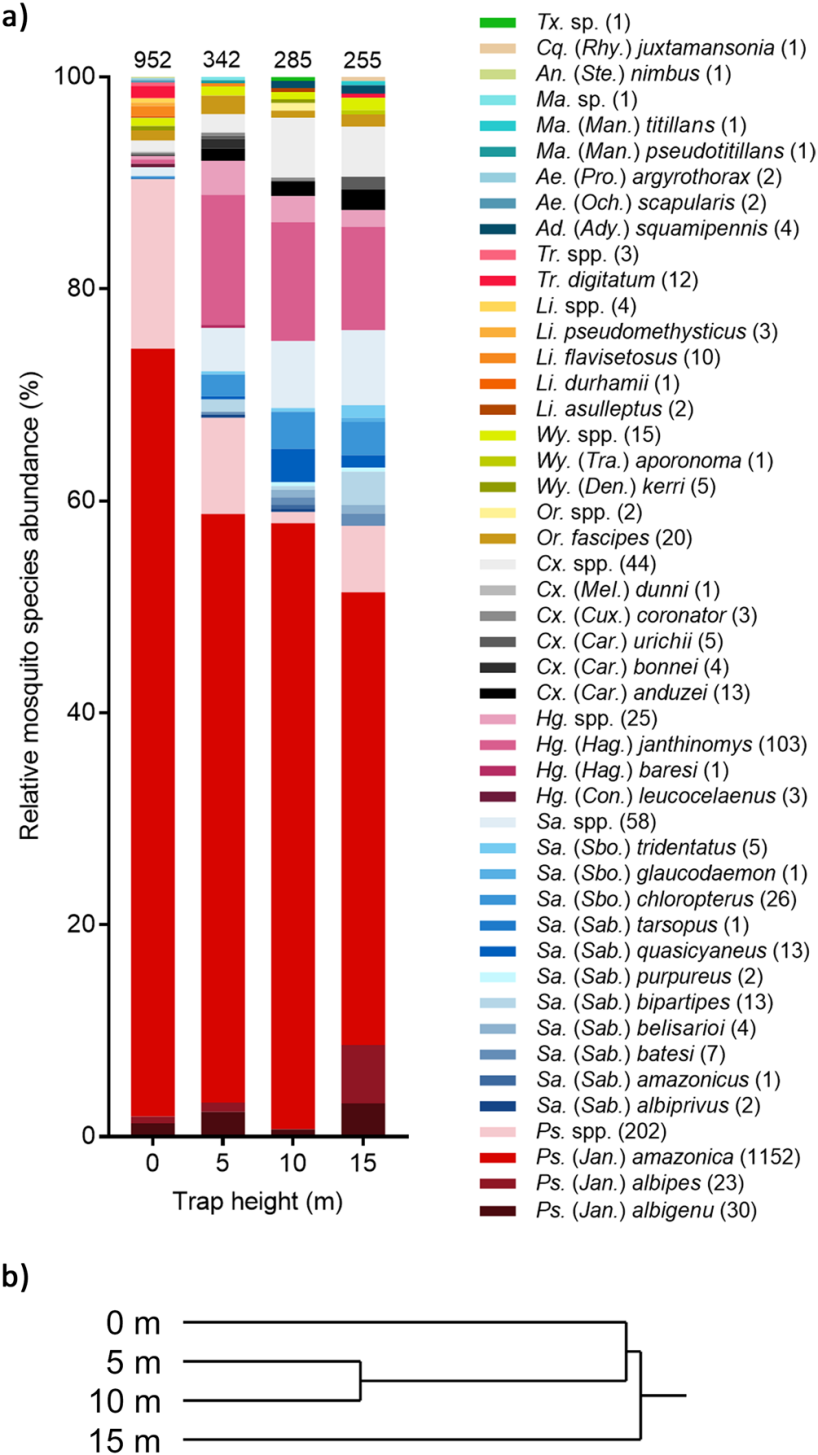
Mosquito species composition by height showing (**a**) the relative abundance of designated species collected using BG-Sentinel traps at each height (N = 93 collections) sampled across three sites combined (N = 372 collections). Number of mosquitoes collected at each height listed at top of bar; number of each species included in parentheses next to species name; genus and subgenus abbreviations follow Walter Reed Biosystematics Unit nomenclature^50^; sp. = single species, spp. = potentially multiple species. (**b**) shows hierarchical clustering of PC1 and PC2 from a principal components analysis of relative species frequency at each height sampled.

Each height was sampled 31 times at each site (N = 124 BG-Sentinel trap collections per site), equivalent to 372 collections across the three sites in total. These collections yielded 1834 mosquitoes representing 14 genera and 37 identified species (Fig. 5), of which 99.8% (1831/1834) were female (Supplementary file: Dataset). All specimens were identified to genus, but damage caused by trap fans, prolonged exposure to heavy rainfall, and high humidity often precluded species-level identifications, particularly of the more ornate species including those within the genus *Sabethes*. The number and percent of mosquitoes identified to species level within each genus were: *Psorophora* (1205/1407, 85.6%), *Sabethes* (75/133, 56.4%), *Haemagogus* (107/132, 81.1%), *Culex* (26/70, 37.1%), *Orthopodoymia* (20/22, 90.1%), *Wyeomyia* (6/21, 28.6%), *Limatus* (16/20, 80%), *Trichoprosopon* (12/15, 80%), *Aedeomyia* (4/4, 100%), *Aedes* (4/4, 100%), *Mansonia* (2/3, 66.7%), *Anopheles* (1/1, 100%), *Coquillettidia* (1/1, 100%), and *Toxorhynchites* (0/1, 0%). Overall, 1479 mosquitoes (80.6% of total collection) were identified to species, and these are summarized in Fig. 5.

*Psorophora amazonica* dominated collections across all sites (77.9% of 1479 mosquitoes identified to species) and was the most abundant species at each height sampled, while *Hg. janthinomys* (7%) was the second most abundant of the identified species. *Sabethes* mosquitoes formed a similar proportion (7.3%) of the total catch and represented the most diverse genus with 11 identified species. However, while *Sa. chloropterus* was the most abundant species within its genus, only 26 specimens were collected, albeit in 23 separate trapping events. Due to the low abundance of target species, analyses focused on occurrence rather than abundance when investigating stratification.

### Differences in mosquito community composition by collection site and height

Linear regression revealed no significant effect of trap height on the mean number of identified species (DF = 1,10, F = 0.47, *P* = 0.51) or on species diversity as measured by the Shannon–Wiener diversity index (F = 3.66, *P* = 0.085). Specimens that were not identified to species level were excluded from these analyses. The Morisita overlap index (Table 2) revealed substantial overlap in community composition at species level between the three sites and mosquito collections were therefore grouped for subsequent analysis of species-specific distributions. There was also substantial overlap among all heights sampled, contrary to expectations that mosquito communities would become more distinct moving from ground level to 15 m. In a principal components analysis of the relative frequency of each species at each height, the first two principal components (PC1 and PC2) explained 78.3% of the variation in the data. A hierarchical cluster analysis of PC1 and PC2 (Fig. 5) revealed that while mosquito communities at all heights overlapped substantially, the community at 0 m was distinct from the communities at 5 and 10 m, which were relatively similar to each other, and more distant from the community at 15 m.

**Table 2:** Morisita overlap index by site and height based on species collected during the study period.

Site	A	B	C	Height	0 m	5 m	10 m	15 m
A	1			0 m	1			
B	0.92	1		5 m	0.95	1		
C	0.88	0.99	1	10 m	0.95	1	1	
				15 m	0.86	0.97	0.96	1

Specimens not identified to species level were excluded from analysis.

### Species-specific distributions

**Figure 6:**
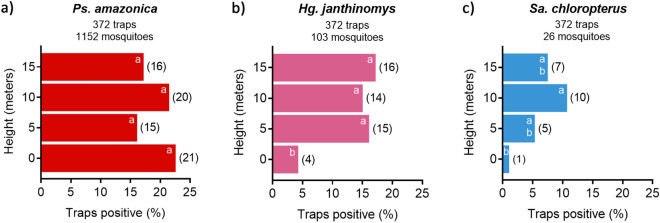
Percent and number of traps positive at each height for (**a**) *Ps. amazonica*, (**b**) *Hg. janthinomys*, and (**c**) *Sa. chloropterus* using data pooled from across the three sites. Different letters within bars indicate significant differences in occurrence between heights for each species; letters indicate within-panel comparisons only. Number of traps positive at each height shown in brackets.

Contingency table analyses revealed no significant effect of height on the occurrence, defined as the percent of traps positive, of *Ps. amazonica* (Pearson’s chi-squared, DF = 3, χ^2^ = 1.791, *P* = 0.62), but there was a significant effect on the occurrence of *Hg. janthinomys* (DF = 3, χ^2^ = 8.72, *P* = 0.033) and *Sa. chloropterus* (DF = 3, χ^2^ = 7.925, *P* = 0.048) (Fig. 6). Pairwise comparisons of height using two-tailed Fisher’s exact tests (exact P values listed in Supplementary Table S3) showed that *Hg. janthinomys* occurred less frequently at ground level than at all other heights (DF = 1, N = 186, *P* < 0.05 for all comparisons), but there was no detectable difference in occurrence when comparing heights above the ground. *Sabethes chloropterus* occurred less frequently at 0 m than at 10 m (DF = 1, N = 186, *P* < 0.01) and there was a similar trend when comparing 0 m with 15 m (DF = 1, N = 186, *P* = 0.06), but this was not significant and no other comparisons were significant for this species (DF = 1, N = 186, *P* > 0.05 for all comparisons). Two-tailed Fisher’s exact tests also revealed no significant difference in the occurrence of *Sa. bipartipes* (DF = 1, N = 372, *P* = 0.072) and *Sa. quasicyaneus* (DF = 1, N = 372, *P* = 0.072) when comparing traps positive at ground level (0 for both species) with those above the ground (11 and 10 traps positive for respective species), although there were only small numbers of traps positive for each species.

### Associations between mosquito distributions and microclimate

**Figure 7:**
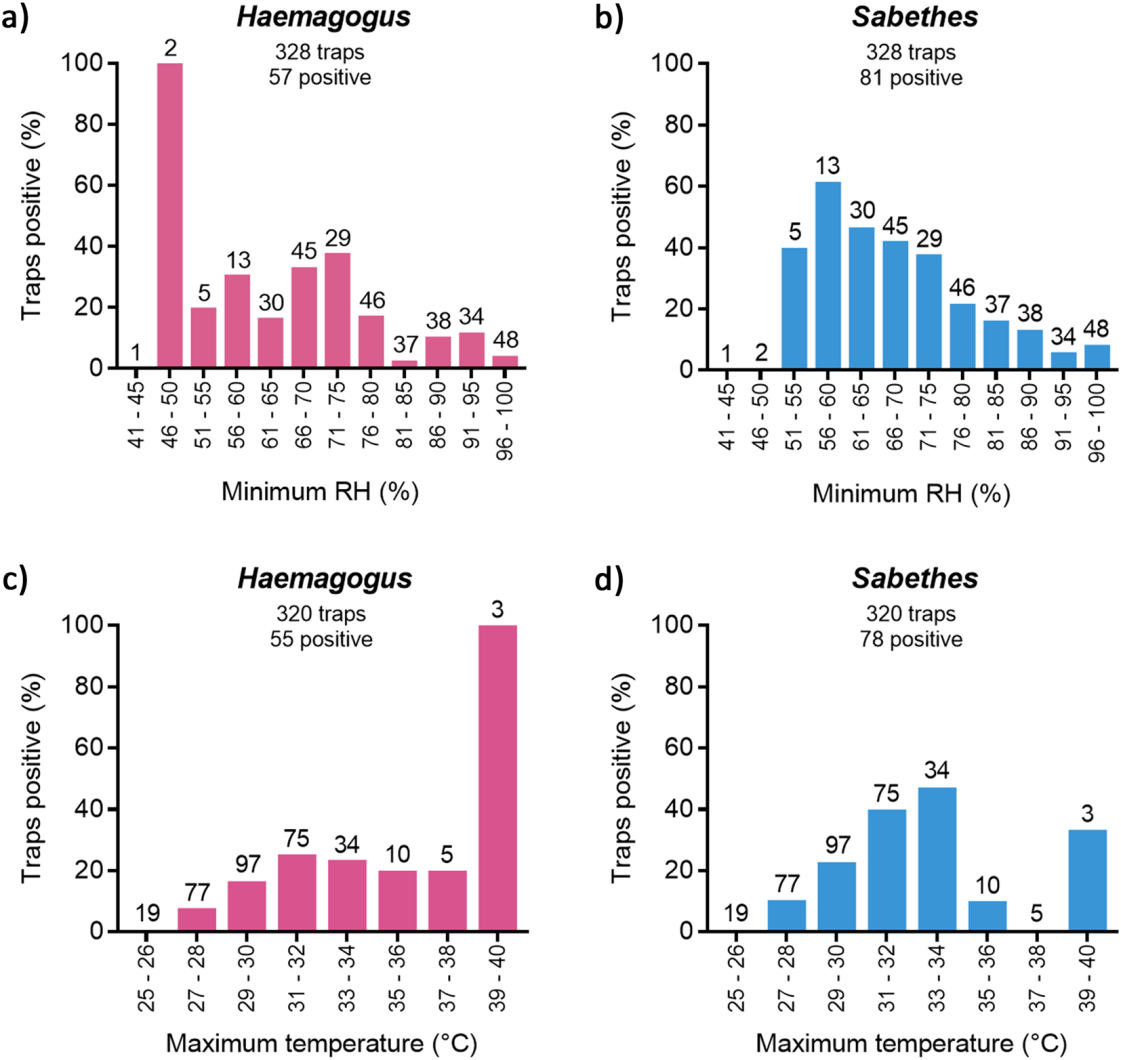
Associations between mosquitoes and microclimate showing percent of traps positive for (**a**) *Haemagogus* and (**b**) *Sabethes* mosquitoes in relation to minimum relative humidity (RH, shown at 5% intervals), and for (**c**) *Haemagogus* and (**d**) *Sabethes* mosquitoes in relation to maximum temperature (shown at 2 °C intervals). Number of traps in each humidity and temperature class shown above each bar.

Nominal logistic regressions revealed negative associations between minimum relative humidity, which was negatively correlated with maximum temperature (Supplementary Table S1), and the occurrence of *Haemagogus* and *Sabethes* mosquitoes within each height sampled. For *Haemagogus*, this was significant at heights of 5 m (DF = 1, χ^2^ = 12.2, *P* = 0.0005) and 10 m (DF = 1, χ^2^ = 3.7, *P* = 0.05), but not at 0 m (DF = 1, χ^2^ = 2.4, *P* = 0.12) or 15 m (DF = 1, χ^2^ = 2.8, *P* = 0.09), although the trend was generally the same. For *Sabethes*, the association was significant at 0 m (DF = 1, χ^2^ = 5.4, *P* = 0.02), 5 m (DF = 1, χ^2^ = 6.4, *P* = 0.01), and 10 m (DF = 1, χ^2^ = 11.5, *P* = 0.0007), but not at 15 m (DF = 1, χ^2^ = 3.0, *P* = 0.08), although again, the trend was generally the same. The overall occurrence of both genera increased in frequency with increasing maximum temperature and decreasing relative humidity (Fig. 7). The negative association with minimum relative humidity within each height was not evident for *Hg. janthinomys* and *Sa. chloropterus*, the most abundant species in each genus, likely due to the small numbers of traps positive for each species. Furthermore, there was no significant relationship between minimum relative humidity and the occurrence of *Psorophora* mosquitoes, or specifically for *Ps. amazonica*, at any of the heights sampled.

### Effects of 7-day cumulative rainfall on the occurrence of mosquitoes

Nominal logistic regressions revealed positive associations between rainfall in the weeks prior to collections and occurrence of key taxa. The occurrence of *Haemagogus* significantly increased with increasing 7-day cumulative rainfall at a lag of 1 week (DF = 1, χ^2^ = 3.9, *P* = 0.0495), while increasing rainfall at a lag of 3 weeks was associated with an increase in the occurrence of *Sabethes* (DF = 1, χ^2^ = 4.4, *P* = 0.0357). Significant increases in the occurrence of *Psorophora* were associated with increasing rainfall at lags of 1 week (DF = 1, χ^2^ = 16.5, *P* < 0.0001) and 4 weeks (DF = 1, χ^2^ = 15.3, *P* < 0.0001). These positive associations were replicated at species level for *Sa. chloropterus* (lag of 3 weeks, DF = 1, χ^2^ = 11.6, *P* = 0.0006) and *Ps. amazonica* (lag of 1 week, DF = 1, χ^2^ = 14.4, *P* = 0.0001; lag of 4 weeks, DF = 1, χ^2^ = 22, *P* < 0.0001), but not for *Hg. janthinomys* (lag of 1 week, DF = 1, χ^2^ = 2.8, *P* = 0.09), although the latter followed a similar trend.

## Discussion

Our study investigated the composition and vertical stratification of mosquito species inside a large forest reserve bordering the city of Manaus, where abrupt edges between urban and forest environments bring humans, mosquitoes, and wildlife into close contact. Such areas increase permeability for the co-occurrence of hosts and bridge vectors, thereby elevating the risk of zoonotic arbovirus exchange^54^. This has the potential to cause outbreaks of novel diseases in urban populations, or, as is the concern for Zika virus in the Americas, lead to spillback and establishment of sylvatic cycles of transmission that could result in an enduring threat to human health^1,55^. Understanding the ecology and behavior of potential bridge vectors is, therefore, critical to understanding the risk of spillover and spillback.

We demonstrated that, as expected^25,27,56^, microclimate varied among forest strata, with the forest floor being cooler and more humid than at elevated heights, and with temperature increasing and humidity decreasing with increasing height. Despite this, we did not detect differences in the mean number (richness) of mosquito species, species diversity, or the overlap of mosquito communities based on the Morisita index. However, a principal components analysis did reveal a hierarchy in the structure of the mosquito community, which changed progressively with increasing height. Confalonieri and Costa Neto^57^ found that species diversity decreased with increasing height when sampling using handheld nets and CDC light traps at the Caxiuanã National Forest in Pará State, Brazil, but that differences were only significant between 0 and 8 m. In the same study, the composition of mosquito communities was found to be quite distinct between 0 and 30 m. Brant et al.^58^ also encountered significant differences in community composition between the ground and canopy (10–20 m) while investigating the stratification of mosquito vectors of zoonotic malaria in Borneo collected using human bait in evenings (18:00–22:00). When considering these findings, it is worth remembering that the use of human bait is inevitably biased towards the collection of human-biting species^59,60^, while BG-Sentinel and CDC light trap collections tend to detect a greater diversity of species^60,61^.

The high number of mosquitoes encountered in BG-Sentinel traps at ground level was driven by the high abundance of *Ps. amazonica*, which is an anthropophilic member of the *Janthinosoma* subgenus^62^ but not a known arbovirus vector. However, flaviviruses, including Ilhéus^63^ and St. Louis encephalitis^64^, have been isolated from *Ps. ferox*, another *Janthinosoma* species. Despite being the most abundant species at all heights sampled, there was no significant difference in the stratification of *Ps. amazonica* based on its occurrence in traps. Its vertical distribution from the forest floor to at least 15 m in height, along with previous observations that it occurs in relatively high abundance at forest edges in urban parks in Manaus^62^, should raise awareness of its potential to serve as a bridge vector, although more information about its feeding habits is needed. At present, little is known about the non-human blood hosts of *Ps. amazonica*, although monkey-baited CDC light traps have been successfully used to collect *Ps. albigenu* and *Ps. ferox*, both *Janthinosoma* species, on the ground and in forest canopy^65^.

In contrast, both *Hg. janthinomys* and *Sa. chloropterus* are known to transmit flaviviruses and may thus be capable of bridging human and sylvatic transmission cycles. YFV has repeatedly been detected in *Hg. janthinomys*, including recently in southeastern Brazil where infection rates of 3.4%^66^ (based on minimum infection rate) and 8.2%^67^ have been reported. Fewer YFV detections and lower infection rates have been reported in *Sa. chloropterus*, which is considered an important secondary vector^66^. The acrodendrophilic habits of these species are well documented and both showed significant differences in stratification in this study. Both were more likely to be present above the ground rather than at ground level, but of the two, *Sa. chloropterus* appeared to slightly favor heights above 5 m. Seventy-four percent of traps positive for this species were located at 10 m or higher, while for *Hg. janthinomys*, this value was 61%. The preference for *Hg. janthinomys* and *Sa. chloropterus* to occupy heights above the ground extends evidence that acrodendrophilic species are infrequently encountered at ground level in well-preserved tropical rainforest^20,25^, although several of our traps were situated near treefall gaps.

The significant breakpoint in stratification of *Hg. janthinomys* between 0 and 5 m corresponded with significant differences in minimum relative humidity and maximum temperature between ground level and all elevated heights. There was generally a less pronounced difference in microclimate among the 5, 10 and 15 m strata (Fig. 4), and there was no difference in stratification of this species among these heights. In one of the earliest field studies of *Hg. janthinomys*, Bates^32^ suspected that stratification was related to humidity and reported increases in abundance at ground level when humidity was lowest. This suspicion has often been echoed by others^68–72^, but there has been little empirical evidence to support it. Our results should be interpreted cautiously but suggest that a negative relationship exists between minimum relative humidity and the occurrence of *Haemagogus* and *Sabethes* mosquitoes and this was evident at several of the heights sampled. At heights where the relationship was not significant, results followed a similar trend. Collecting enough target mosquitoes in identifiable condition using BG-Sentinel traps was a challenge throughout the study, but since *Hg. janthinomys* formed 96.3% of the total identified *Haemagogus* catch, it seems reasonable to assume that these findings also apply to this species. Such a negative relationship with humidity would appear to agree with observations that *Haemagogus* and *Sabethes* mosquitoes increase in abundance at ground level in forest clearings and at forest edges^20,26,31,32,34,68^ where humidity is lower and temperature is higher than in undisturbed habitat^19,73^. As Bates^32^ suggested, occurrence at ground level may be further exacerbated during daytime hours when relative humidity is lowest (Fig. 3), increasing the risk of contact with humans and the bridge capacity of these mosquitoes.

Establishing links between rainfall and the appearance of vector species has potential use for modeling arbovirus disease dynamics^74^. We demonstrated that the appearance of *Haemagogus*, *Sabethes*, and *Psorophora* was related to seven-day cumulative rainfall in the weeks prior to collections. The occurrence of *Haemagogus* was associated with rainfall at a lag of 1 week, while *Sabethes*, including *Sa. chloropterus*, was associated with rainfall at a lag of 3 weeks. Some laboratory studies have reported slightly faster egg to adult development times for *Haemagogus* species^75–77^ than for *Sa. chloropterus*^78^, and our results appear to support this pattern under natural conditions. While little is known about the development times of *Ps. amazonica*, other *Janthinosoma* species undergo fairly rapid life cycles^79–82^. For *Ps. varipes*, this can be completed in around 16–19 days at 27 °C in the laboratory^79^, while successive broods of *Ps. ferox* have been observed to emerge about a month apart in Florida under natural conditions^80^. The association of *Ps. amazonica* with rainfall at lags of 1 and 4 weeks may, therefore, reflect the emergence of successive generations of this species resulting from a similar short life cycle. These findings indicate interesting variations in the life history of these taxa that should be examined more closely in the laboratory and in nature.

Our study had several limitations that may provide scope for further research. First, we terminated sampling at the end of the rainy season when mosquito abundance declines sharply. Extending our sampling window may have provided information about vector species persistence into the dry season, which is relevant to the maintenance cycles of sylvatic arboviruses. There is only limited evidence for transovarial transmission of YFV by *Haemagogus* species^83,84^, and while it has been shown that *Hg. janthinomys*^7^ and *Sa. chloropterus*^29^ may persist through the dry season, it is not known whether they remain sufficiently abundant to maintain transmission. Dry season sampling may also have provided further information about seasonal changes in stratification that have been observed for these species^29,32^. While BG-Sentinel trap collections should give reliable estimates of relative mosquito abundance, inferences about the absolute abundance of *Hg. janthinomys*, *Sa. chloropterus*, and *Ps. amazonica* are limited. It is not known how attractive BG-Sentinel traps are to these species, which were designed for the collection of *Aedes* species mosquitoes^85^. Similar collections using human bait, or a direct comparison of these methods would be worthwhile. Lastly, BG-Sentinel trap collections yielded insufficient numbers of identifiable *Hg. janthinomys* and *Sa. chloropterus* to perform microclimate analyses at species level, thus we were limited to analyzing associations of genera with microclimate.

The risk of arbovirus spillback will ultimately be influenced by the frequency of interactions between susceptible hosts and competent vectors in the presence of pathogens, and the stratification of mosquitoes relative to humans and monkeys may provide an indication of interactions most likely to take place. The higher occurrence and greater abundance of *Hg. janthinomys* relative to *Sa. chloropterus* suggests that the former may be the most likely of the known vectors to encounter humans at ground level, although neither was frequently encountered in BG-Sentinel traps on the forest floor. Both *Hg. janthinomys* and *Sa. chloropterus* have previously been collected at higher heights than sampled in this study^25,31^ and may, therefore, provide a route of arbovirus transmission between humans and monkeys active higher in the forest canopy. Of the monkeys present at the Ducke reserve, *Ateles paniscus*^86,87^ and *Alouatta* species^86,88^ are most frequently encountered above 20 m but seldom occur at forest edges. Both *Chiropotes*^86^ and *Pithecia* species^86,89^ often occur above 15 m, but the latter also occurs below 15 m and is more likely to enter edge habitats. However, *Sapajus apella*^86,90,91^ and *Saguinus bicolor*^92,93^ are the species most likely to be found below 15 m and at forest clearings and forest edges. It is in these edge habitats, where humans and these mosquito and monkey species coexist, that interactions between hosts and vectors are perhaps most likely to take place, creating opportunities for spillback of ZIKV and other mosquito-borne viruses.

## Conclusions

The vertical stratification of *Hg. janthinomys* and *Sa. chloropterus* suggests that both are infrequently encountered at ground level in the Ducke reserve based on BG-Sentinel trap collections. However, the occurrence of both genera may increase at higher temperature and lower relative humidity, a characteristic feature of forest edges. These are habitats where monkeys such as *Saguinus bicolor* and *Sapajus apella* occupy the lower forest strata and are areas at risk of zoonotic arbovirus exchange when humans exist in close contact. Our previous work has shown that the spatial distribution of *Ps*. *amazonica* extends from the forest interior to its edge. The dominance of this species at all heights sampled during this study provides further evidence that its distribution overlaps with human and sylvatic arbovirus hosts in Manaus, where it should also be investigated as a potential bridge vector.

## Supplementary information

Supplementary Information 1.

Supplementary Information 2.

## Data Availability

All data generated or analysed during this study are included in this published article (and its Supplementary Information files).
