# Supplementary material for: The vertical stratification of potential bridge vectors of mosquito-borne viruses in a central Amazonian forest bordering Manaus, Brazil
Source: Sci Rep. 2020 Oct 26;10:18254. doi: 10.1038/s41598-020-75178-3 (PMC7589505; doi:10.1038/s41598-020-75178-3)
Supplement: Supplementary file 2 — Supplementary Information 2. [file 41598_2020_75178_MOESM2_ESM.pdf]

Supplementary Information

The vertical stratification of potential bridge vectors of mosquito-borne viruses in a central Amazonian forest bordering Manaus, Brazil

Adam Hendy, Eduardo Hernandez-Acosta, Danielle Valério, Claudia Mendonça, Edson Rodrigues Costa, José Tenaçol Andes Júnior, Flamarion Prado Assunção, Vera Margarete Scarpassa, Marcelo Gordo, Nelson Ferreira Fé, Michaela Buenemann, Marcus Vinícius Guimarães de Lacerda, Kathryn A. Hanley, Nikos Vasilakis

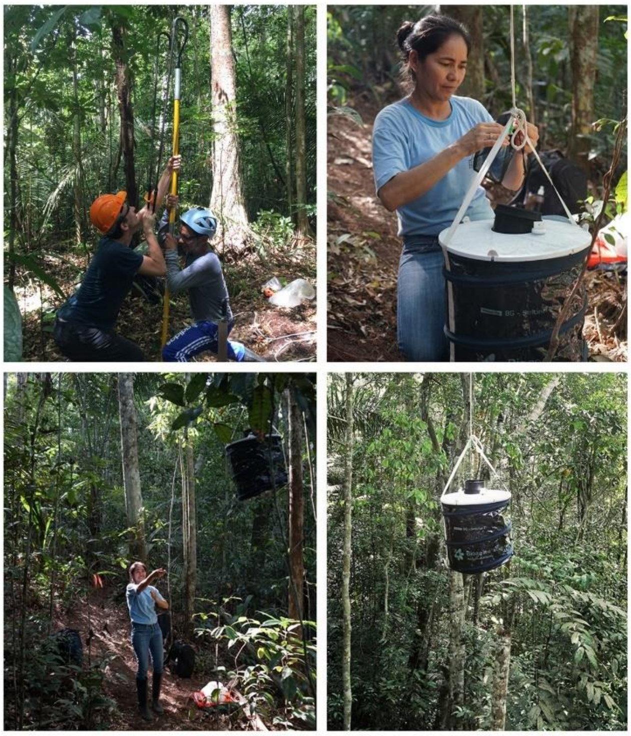

Fig S1. BG-Sentinel trap placement in a tree using BigShot slingshot. Top L-R: lead weight and fishing line being launched over a branch to which a rope is attached; trap being prepared. Bottom L-R: trap being raised to desired height; trap hanging at 10 m from a tree.

Table S1. Pairwise Spearman's rank analysis of microclimate variables and trap height, showing results of significant correlations. Black text = positive correlation; red text = negative correlation; Spearman's correlation coefficients (Rho) included in brackets.

	Total rain	Early rain	Late rain	Daytime rain	Min temp	Max temp	Mean temp	Daytime mean temp	Temp range	Min humidity	Max humidity	Mean humidity	Daytime mean humidity	Humidity range	Trap height meters
Total rain															
Early rain	<0.0001 (0.3316)														
Late rain	<0.0001 (0.5982)	<0.0001 (0.2718)													
Daytime rain	<0.0001 (0.5854)	<0.0001 (0.6756)	<0.0001 (0.8070)												
Min temp	<0.0001 (-0.3669)		<0.0001 (-0.3356)	0.0003 (-0.2002)											
Max temp	<0.0001 (-0.3769)	<0.0001 (-0.3121)	<0.0001 (-0.4499)	<0.0001 (-0.4679)	<0.0001 (0.3884)										
Mean temp	<0.0001 (-0.5821)	0.0001 (-0.2130)	<0.0001 (-0.5799)	<0.0001 (-0.5050)	<0.0001 (0.6998)	<0.0001 (0.7785)									
Daytime mean temp	<0.0001 (-0.5583)	<0.0001 (-0.2376)	<0.0001 (-0.6156)	<0.0001 (-0.5397)	<0.0001 (0.6138)	<0.0001 (0.8395)	<0.0001 (0.9719)								
Temp range	<0.0001 (-0.2639)	<0.0001 (-0.3509)	<0.0001 (-0.3647)	<0.0001 (-0.4336)		<0.0001 (0.9403)	<0.0001 (0.5899)	<0.0001 (0.6853)							
Min humidity	<0.0001 (0.3254)	<0.0001 (0.3084)	<0.0001 (0.4422)	<0.0001 (0.4685)	0.0001 (-0.2152)	<0.0001 (-0.8811)	<0.0001 (-0.6801)	<0.0001 (-0.7712)	<0.0001 (-0.8783)						
Max humidity	<0.0001 (0.3720)	<0.0001 (0.2606)	0.0001 (0.2096)	<0.0001 (0.2836)	<0.0001 (-0.4168)	<0.0001 (-0.3745)	<0.0001 (-0.5558)	<0.0001 (-0.4977)	<0.0001 (-0.2635)	<0.0001 (0.3823)					
Mean humidity	<0.0001 (0.4627)	<0.0001 (0.3402)	<0.0001 (0.4792)	<0.0001 (0.5028)	<0.0001 (-0.3900)	<0.0001 (-0.8064)	<0.0001 (-0.8242)	<0.0001 (-0.8706)	<0.0001 (-0.7338)	<0.0001 (0.8728)	<0.0001 (0.6541)				
Daytime mean humidity	<0.0001 (0.4353)	<0.0001 (0.3159)	<0.0001 (0.4992)	<0.0001 (0.5034)	<0.0001 (-0.3748)	<0.0001 (-0.8346)	<0.0001 (-0.8126)	<0.0001 (-0.8808)	<0.0001 (-0.7711)	<0.0001 (0.9153)	<0.0001 (0.5645)	<0.0001 (0.9861)			
Humidity range	<0.0001 (-0.2855)	<0.0001 (-0.2707)	<0.0001 (-0.4576)	<0.0001 (-0.4604)	0.02 (0.1300)	<0.0001 (0.8327)	<0.0001 (0.5863)	<0.0001 (0.6877)	<0.0001 (0.8559)	<0.0001 (-0.9660)	<0.0001 (-0.2261)	<0.0001 (-0.7655)	<0.0001 (-0.8244)		
Trap height meters					0.01 (-0.1411)	<0.0001 (0.4091)	0.03 (0.1193)	0.0002 (0.2058)	<0.0001 (0.4875)	<0.0001 (-0.4615)		<0.0001 (-0.2972)	<0.0001 (-0.3388)	<0.0001 (0.4841)	

Total rain = Total rain (in mm) during sampling period based on INMET weather station A101-Manaus; **Early rain** = Early rain (08:00 - 13:00) (in mm) on the day the trap was set; **Late rain** = Late rain (14:00 - 17:00) (in mm) on the day the trap was set; **Daytime rain** = Daytime rain (08:00 - 17:00) (in mm) on the day the trap was set**; Min temp** = Minimum temperature (°C) recorded by iButton during the sampling period; **Max temp** = Maximum temperature (°C) recorded by iButton during the sampling period; **Mean temp** = Mean temperature (°C) recorded by iButton during the sampling period; **Daytime mean temp** = Mean temperature (°C) recorded by iButton for all daytime hours (06:00 - 18:00) during the sampling period; **Temp range** = Difference between minimum and maximum temperatures (°C) recorded by iButton during the sampling period; **Min humidity** = Minimum relative humidity (%) recorded by iButton during the sampling period; **Max humidity** = Maximum relative humidity (%) recorded by iButton during the sampling period; **Mean humidity** = Mean relative humidity (%) recorded by iButton during the sampling period; **Daytime mean humidity** = Mean relative humidity (%) recorded by iButton for all daytime hours (06:00 - 18:00) during the sampling period; **Humidity range** = Difference between minimum and maximum relative humidity (%) recorded by iButton during the sampling period; **Trap height meters** = Trap height in meters.

Table S2. Results of Kruskal-Wallis tests showing a significant effect of height on minimum relative humidity and maximum temperature, and post-hoc Wilcoxon Each Pair analyses comparing heights for each parameter. **DF** = degrees of freedom; significant P values (**P =**) for post-hoc analyses highlighted in bold.

Parameter	DF	Kruskal-Wallis, P = (χ^2)	Heights compared	Wilcoxon, P =
Minimum relative humidity	3	<0.0001 (75.5750)	0 m - 5 m	**<0.0001**
			0 m - 10 m	**<0.0001**
			0 m - 15 m	**<0.0001**
			5 m – 10 m	0.1303
			5 m – 15 m	**0.0058**
			10 m – 15 m	0.2920
Maximum temperature	3	<0.0001 (55.8249)	0 m - 5 m	**0.0003**
			0 m - 10 m	**<0.0001**
			0 m - 15 m	**<0.0001**
			5 m – 10 m	**0.0393**
			5 m – 15 m	**0.0010**
			10 m – 15 m	0.1811

Table S3. Results of Pearson's chi-squared tests showing a significant effect of height on the occurrence of *Haemagogus janthinomys* and *Sabethes chloropterus*, and post-hoc two-tailed Fisher's Exact tests comparing heights for each species. **DF** = degrees of freedom; significant P values (**P =**) for post-hoc analyses highlighted in bold.

Species	DF	Pearson's, P = (χ2)	Heights compared	Fisher's, P =
Haemagogus janthinomys	3	0.0333 (8.720)	0 m - 5 m	**0.0136**
			0 m - 10 m	**0.0233**
			0 m - 15 m	**0.0078**
			5 m - 10 m	1
			5 m - 15 m	1
			10 m - 15 m	0.84
Sabethes chloropterus	3	0.0476 (7.925)	0 m - 5 m	0.2111
			0 m - 10 m	**0.0095**
			0 m - 15 m	0.0645
			5 m - 10 m	0.2813
			5 m - 15 m	0.7668
			10 m - 15 m	0.6120
